# Relationships between population density, fine-scale genetic structure, mating system and pollen dispersal in a timber tree from African rainforests

**DOI:** 10.1038/hdy.2015.101

**Published:** 2015-12-23

**Authors:** J Duminil, K Daïnou, D K Kaviriri, P Gillet, J Loo, J-L Doucet, O J Hardy

**Affiliations:** 1 Bioversity International, Forest Genetic Resources Programme, Sub-Regional Office for Central Africa, Yaoundé, Cameroon; 2 Service Evolution Biologique et Ecologie, Faculté des Sciences, Université Libre de Bruxelles, Brussels, Belgium; 3 Département BIOSE, Gestion des Ressources forestières, Foresterie Tropicale, Gembloux Agro-Bio Tech, Université de Liège, Gembloux, Belgium; 4 Nature+ asbl, Wavre, Belgium; 5 Laboratoire de Génétique, Amélioration des Plantes et Biotechnologie, Faculté des Sciences, Université de Kisangani, Kisangani, Democratic Republic of Congo; 6 Bioversity International, Headquarters, Via dei Tre Denari, Maccarese (Fiumicino), Rome, Italy

## Abstract

Owing to the reduction of population density and/or the environmental changes it induces, selective logging could affect the demography, reproductive biology and evolutionary potential of forest trees. This is particularly relevant in tropical forests where natural population densities can be low and isolated trees may be subject to outcross pollen limitation and/or produce low-quality selfed seeds that exhibit inbreeding depression. Comparing reproductive biology processes and genetic diversity of populations at different densities can provide indirect evidence of the potential impacts of logging. Here, we analysed patterns of genetic diversity, mating system and gene flow in three Central African populations of the self-compatible legume timber species *Erythrophleum suaveolens* with contrasting densities (0.11, 0.68 and 1.72 adults per ha). The comparison of inbreeding levels among cohorts suggests that selfing is detrimental as inbred individuals are eliminated between seedling and adult stages. Levels of genetic diversity, selfing rates (∼16%) and patterns of spatial genetic structure (*Sp* ∼0.006) were similar in all three populations. However, the extent of gene dispersal differed markedly among populations: the average distance of pollen dispersal increased with decreasing density (from 200 m in the high-density population to 1000 m in the low-density one). Overall, our results suggest that the reproductive biology and genetic diversity of the species are not affected by current logging practices. However, further investigations need to be conducted in low-density populations to evaluate (1) whether pollen limitation may reduce seed production and (2) the regeneration potential of the species.

## Introduction

The population density of reproductive individuals (‘population density' for short) is central in the context of sustainability of tree resources under selective logging practices. Harvesting implies, among other effects, a reduction of population density with potential prejudicial consequences on reproduction, regeneration and genetic diversity (Degen *et al.*, 2006; Sebbenn *et al.*, 2008; Wernsdörfer *et al.*, 2011; de Lacerda *et al.*, 2013). Plant reproduction, and in particular the extent of gene flow (defined here as the sharing of alleles through mating among individuals), depends on the mating system (from selfing to outcrossing) and the pollen and seed dispersal abilities (Wilcock and Neiland, 2002; Levin *et al.*, 2003; Duminil *et al.*, 2009). A reduction of population density may limit the amount of pollen available for outcrossing, which could reduce seed production (pollen limitation) in obligate outcrossing species and/or increase selfing rate in self-compatible hermaphrodite species (Naito *et al.*, 2008). In the latter case, the quality of seeds would be reduced if inbreeding depression occurs, a phenomenon commonly reported in tree species (Charlesworth, 2003; Duminil *et al.*, 2009). However, the impact of population density reduction on animal-pollinated species is highly variable as it depends on a number of factors, including the behaviour of pollinators (Karron *et al.*, 1995; Ghazoul, 2005). In a number of species, a reduction of population density has been shown to be counterbalanced by a change in pollinator behaviour that allows higher pollen dispersal distance (Hardy *et al.*, 2006; Carneiro *et al.*, 2011). Another potential long-term impact of a demographic reduction is a higher rate of loss of rare alleles, reducing the population adaptive potential (Beardmore *et al.*, 2014). Here again, this impact might be counterbalanced if effective pollen dispersal distances increase at lower density. We could imagine, however, that pollen dispersal distances are smaller below some density threshold where a disruption of pollen flow between individuals would occur because of the unavailability of mates (Forsyth, 2003; de Waal *et al.*, 2015).

The effect of population density reduction on plant species' reproductive biology and genetic diversity has notably been addressed by studying the impact of logging on timber tree species. Indeed, logging results in a reduction of population density. However, logging may also affect reproductive biology through the environmental changes it induces (for example, canopy openings may affect wind movements and pollinators' population size or behaviour, cause stress or, alternatively, favour the regeneration of light-demanding species). Studies addressing the impact of logging have provided contradictory results. Most studies have demonstrated a limited impact of logging on timber species reproductive biology and genetic diversity after one event of logging only (one cutting cycle) (Cloutier *et al.*, 2007; Silva *et al.*, 2008; Carneiro *et al.*, 2011). However, modelling approaches have demonstrated that multiple cycles of logging can have a detrimental impact on genetic diversity and demography of harvested tree species (Degen *et al.*, 2006; Sebbenn *et al.*, 2008; Wernsdörfer *et al.*, 2011; de Lacerda *et al.*, 2013). Overall, these studies demonstrate that each species is unique. Accordingly, sustainable forest management practices must be species specific, taking into account the complex relationship between tree density and species reproductive biology. Unfortunately, in Africa, the biology of most tropical species is poorly documented because of the large number of species and logistical difficulties in conducting fieldwork in tropical forests.

Ideally, the impact of logging on population dynamics should be evaluated by a diachronic approach characterizing patterns of gene flow and genetic diversity several years before and after logging (Cloutier *et al.*, 2007; Silva *et al.*, 2008; Carneiro *et al.*, 2011). Alternatively, the impact of logging can be investigated indirectly through a synchronic approach by comparing gene flow patterns of populations with contrasting densities (Piotti *et al.*, 2012; Inza *et al.*, 2012; Ojeda-Camacho *et al.*, 2013; Fageria and Rajora, 2013). Although indirect, the latter approach has the advantage of focussing on one factor (population density) that is affected by logging. In contrast, the synchronic approach should be less affected by such confounding factors when assessing the impact of population density on processes (rather than patterns) such as selfing and pollen dispersal.

Species reproductive biology can be characterized by genetic markers, using either direct or indirect approaches. Direct methods (see, for example, Marshall *et al.*, 1998) estimate contemporary gene flow by conducting parentage analysis, whereas indirect methods estimate either contemporary pollen flow by characterizing the spatial genetic structure of pollen clouds (Robledo-Arnuncio *et al.*, 2007) or historical gene flow by characterizing the spatial genetic structure of adults (see, for example, Vekemans and Hardy, 2004). Although direct methods are generally preferable because they do rely on fewer model assumptions than the indirect approaches (Vekemans and Hardy, 2004), they are also more difficult to set up (availability of highly polymorphic markers, difficulties associated with exhaustive sampling in natural tropical forests and so on).

We applied both indirect and direct methods to assess gene flow and mating patterns on three different populations of *Erythrophleum suaveolens*, an important timber tree species in Central Africa, commonly known as ‘tali'. Here we compare the reproductive biology and genetic diversity of populations that present contrasting population densities. As outlined before, this methodology allows investigation of one aspect of the potential impact of logging on population dynamics: population density reduction. We can thus expect lower genetic diversity in a low-density population if gene flow does not compensate for an increase in local genetic drift. In the present paper, we analysed whether a lower population density was associated with: (1) lower genetic diversity, (2) stronger spatial genetic structure due to higher local genetic drift, (3) higher selfing rate and inbreeding, (4) fewer pollen donors contributing to the pollination of each mother tree and (5) a change of pollen dispersal distances. Theoretically, our expectations (1) to (4) should hold if the extent of pollen dispersal is unaffected by population density, or decreases at low density, whereas an increase in pollen dispersal distances under low population density (relationship (5)) might result in a lack of evidence to support any of the other expectations.

## Materials and methods

### Species description

*E. suaveolens* (Guill. et Perr.) Brenan (syn. *E. guineense* G. Don.) is an important timber species (up to 40 m in height) that belongs to the Fabaceae–Caesalpinioideae. It has widespread distribution in tropical Africa, occuring east–west from Senegal to Sudan and Kenya, and southward to Mozambique and Zimbabwe (Aubréville, 1959, 1970). Within its Central Africa distribution, the species inhabits inland semi-evergreen and evergreen rainforests (Duminil *et al.*, 2010). Individuals are hermaphrodites (Aubréville, 1970), producing racemes up to 12-cm long that bear small-size yellowish white to greenish yellow flowers. The mean flowering time of *E. suaveolens* individuals is ca. 2 months, whereas the mean flowering time of the population is ca. 4 months (F Feteke, Gembloux Agro-Bio Tech, unpublished results). Pollen dispersal is probably assisted by small insects as suggested by the size of the flowers and by observations in the congeneric species *Erythrophleum fordii* (Zhu *et al.*, 2009). Pods do not present any morphological device for dispersal, suggesting predominant barochory. Seeds in fresh pods are surrounded by a mucilage (Guion, 2011) that might have a nutritive value leading to secondary seed dispersal by animals. This is further supported by the presence of seeds in the faeces of primates (Poulsen *et al.*, 2001), including gorilla (Petre *et al.*, 2015). The soil seedbank of the species is important and seeds probably remain viable for ⩾2 years (Daïnou *et al.*, 2011). The species presents a bell-shaped distribution of diameter size classes (Supplementary Material 1 online), with little representation of low- and high-diameter classes (Kouadio *et al.*, 2014), suggesting reduced natural regeneration in mature closed-canopy forests.

As for all rainforest timber species from Central Africa, *E. suaveolens* is harvested by selective logging (Ruiz Pérez *et al.*, 2005). Logging carried out in Cameroon, Democratic Republic of Congo (DRC) and Gabon does not follow exactly the same standards (Perthuisot and Durrieu De Madron, 2008).The rate of logging is calculated taking into account: (1) a cutting cycle (20 to 30 years according to the country or the logging company); (2) a minimum recovery rate (ratio of the number of trees that will reach the minimum diameter-felling limit at the end of the cutting cycle to the number of trees that already attained the diameter-felling limit before the cutting cycle; the threshold for recovery rate per species was fixed at 40% in Gabon and DRC and 50% in Cameroon); and (3) a minimum diameter-felling limit (fixed at 70 cm diameter at breast height (DBH) in Gabon and 50 cm DBH in Cameroon and DRC).

### Sampling

**Figure 1:**
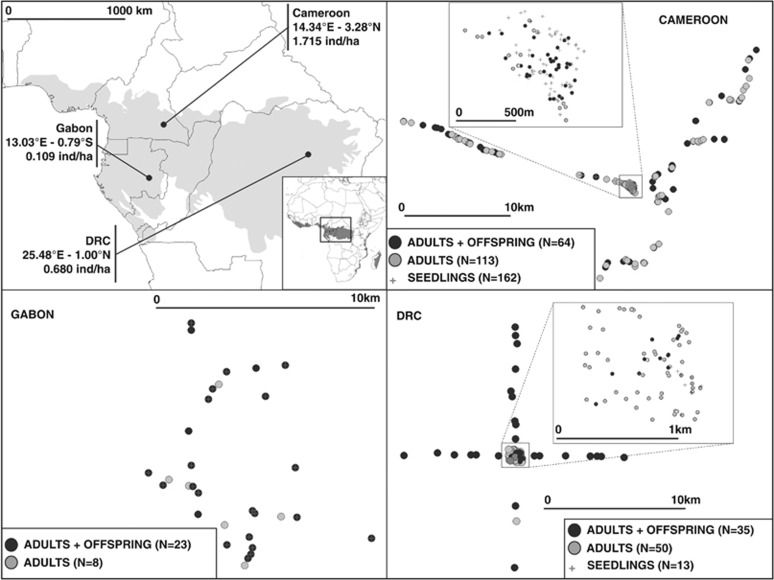
Localization of the three populations and sampling scheme in each population. Upper left: localization of the three studied populations of *Erythrophleum suaveolens* in the Central African forest (grey shaded area represents the potential rainforest area). Upper right: sampling scheme in Cameroon. An exhaustive sampling was conducted in a 47.5-ha plot as represented in the embedded figure. Lower left: sampling scheme in Gabon. Lower right: sampling scheme in DRC. An exhaustive sampling was conducted in a 100-ha plot as represented in the embedded figure.

Adult trees are defined as individuals that can contribute to pollination (DBH above 30 cm according to Kouadio, 2009). Leaves or cambium of adult trees and offspring (seeds and leaves of seedlings) were collected in three different populations from Central Africa (Figure 1 and Supplementary Material 2 online). The first population is located in Cameroon within the Forest Stewardship Council (FSC)-certified ‘Pallisco' logging concession (East province; mean coordinates: 14.34°E, 3.28°N). The second population is located in DRC within the ‘Compagnie de Transport et d'Exploitation Forestière (COTREFOR)' logging concession (Orientale province; mean coordinates: 25.48°E, 1.00°N). The last population is located in Gabon within the FSC-certified ‘Precious Woods' logging concession (Ogooué-Lolo province; mean coordinates: 13.03°E, 0.79°S). Sampling was done in January–March 2012 in Cameroon and Gabon and in August 2013 in DRC.

These populations present contrasting tree densities (*D*, population density measured for DBH >30 cm), with the highest density found in Cameroon (1.72 ind ha^−1^), an intermediate density in DRC (0.68 ind ha^−1^) and the lowest density in Gabon (0.11 ind ha^−1^). In Cameroon and DRC we sampled a maximum number of individuals (adults, offspring) in 47.5 and 100 ha plots respectively and along three or four 4.5 to 11 km long transects departing from each plot (Figure 1). These two populations were never logged. In contrast, the population from Gabon has been logged twice, first, 15 years ago primarily targeting *Aucoumea klaineana*. Although *E. suaveolens* was not logged at that time, the opening of the canopy induced by this logging event has probably favoured the regeneration of *E. suaveolens* that would explain the relatively higher representation of low diameter classes in this population compared with the other two populations (Supplementary Material 1 online). Second, 60 to 70% of individuals of *E. suaveolens* with a DBH >70 cm were logged in the sampling zone between 2010 and 2011. This recent logging event has favoured species regeneration as attested by the high number of available seedlings (see below) in contrast to the other two populations.

In Gabon, where population density was very low, we sampled as many individuals as possible in a zone of ∼7000 ha. In total 177, 88 and 31 adult trees were sampled respectively in Cameroon, DRC and Gabon, among which 72, 33 and 23 were mother trees from which offspring families were also sampled. Seeds or seedlings' leaves were collected on the ground below mother trees (Supplementary Material 2 online). Seeds were predominantly available in Cameroon and DRC, whereas seedlings were predominantly available in Gabon. Most progeny families were composed of seven or eight offspring (Supplementary Material 3 online). Note that this difference in sampling (seeds versus seedlings) could have consequences for the estimation of selfing rate if an elimination of selfed individuals occurs between the seed and seedlings stage.

### Genetic diversity and consanguinity characterization

DNA extraction and genotyping of nine microsatellite markers were carried out as described in Duminil *et al.* (2011). Using SPAGeDi v.1-5 (Hardy and Vekemans, 2002), we estimated for each population and cohort (adults, seedlings, seeds) (1) the effective number of alleles (*NA*_E_) following Nielsen *et al.* (2003); (2) the allelic richness expressed as the expected number of alleles among *k* gene copies (*A*_R_(*k*=24)); (3) the expected heterozygosity (gene diversity) corrected for sample size (*H*_E_) (Nei, 1978); (4) the observed heterozygosity (*H*_O_); and (5) the inbreeding coefficient (*F*_IS_).

Differences in genetic diversity parameters between cohorts and populations were tested using an analysis of variance procedure in R (R Core Team, 2013). The different genetic parameters (*NA*_E_, *A*_R_, *H*_E_, *H*_O_, *F*_IS_) were estimated for each locus, cohort and population. The parameters were compared among populations for adults only accounting for the locus effect. Then, for each population, the parameters were compared among cohorts accounting for the locus effect.

The presence of null alleles has previously been demonstrated (Duminil *et al.*, 2011). As null alleles cause a bias in the proportion of heterozygotes used to estimate Wright's inbreeding coefficient *F*, we also used INEST (Chybicki and Burczyk, 2009) under a Population Inbreeding Model to estimate for each cohort and population *F*_(null)_, an estimator of inbreeding coefficient that removes the bias caused by null alleles. In each population we tested whether the level of inbreeding was significantly different between cohorts by applying unpaired *t*-tests on (*H*_0_) per individual (proportion of heterozygous loci) in each cohort, considering only individuals genotyped for at least seven out of nine loci. Under inbreeding depression, if inbred (that is, less heterozygous) individuals are counter selected at early life stages, we expect to observe more heterozygosity in adults than in seeds and/or seedlings.

### Fine-scale spatial genetic structure

Spatial genetic structure (SGS) of adult trees was assessed for each population. Pairwise kinship coefficients (*F*_*ij*_) between individuals (Loiselle *et al.*, 1995) and 95% confidence intervals were estimated at regular geographical distance intervals using SPAGeDi v.1-4c (Hardy and Vekemans, 2002). SGS was tested by permuting randomly the position of the individuals (10 000 randomizations). Estimates of the *Sp* statistic (a synthetic measure of SGS strength) were obtained for each population from the slope of the regression of pairwise kinship coefficient on ln(distance) and the mean pairwise kinship coefficient measured at the first distance class (*F*_1_), following Vekemans and Hardy (2004).

Additionally, assuming drift–dispersal equilibrium, we estimated the neighbourhood size and the gene dispersal distance σ_g_ for each population relying on SGS patterns following the procedure described in Hardy *et al.* (2006). The principle of the method is that *F*_*ij*_ is expected to decay linearly with the ln(distance) at a rate inversely proportional to the product *D*_E_.*σ*_g_^2^, at least for a distance range between *σ*_g_ and ca. 20 *σ*_g_, where *D*_E_ is the effective density of reproductive individuals and *σ*_g_^2^ is the axial variance of gene dispersal distance between two generations. As the regression must be performed on a distance interval depending on the parameter to estimate (*σ*_g_), an iterative procedure is implemented in SPAGeDi and should converge only if data were sampled at an adequate spatial scale (Hardy *et al.*, 2006). Different values of *D*_E_ were tested, considering 1/2, 1/4 and 1/8 of the adult densities to account for the lifetime variation in reproductive success among adult trees (Hardy *et al.*, 2006). Thus, we tested respectively for Cameroon, DRC and Gabon the following effective densities: 0.850/0.425/0.212, 0.340/0.170/0.085 and 0.054/0.027/0.013 (ind ha^−1^). Approximate standard errors are obtained by jackknifing over loci.

### Assignments of offspring to mothers

As a prerequisite to mating system and pollen pool analyses, given the potential for secondary seed dispersal, we first tested whether the genotypes of candidate mothers and seeds/seedlings that were collected below them were compatible with a mother–offspring relationship. We conducted a maternal analysis using CERVUS (Marshall *et al.*, 1998). CERVUS uses a maximum likelihood approach and assigns maternity according to the highest likelihood (LOD score). Simulations were conducted to estimate the critical values of LOD score required to assign maternity with a given degree of confidence (80 and 95% confidence levels). The following simulation parameters were applied to define the confidence level of maternity analysis assignment: 10 000 simulated mating events; all adults in a population were considered to be candidate mothers; individuals were typed at a minimum of five loci; 90% of candidate mothers were sampled; and a genotyping error rate of 0.1. Only offspring correctly assigned to the expected mother (mother localized above collected offspring) were used in the following steps. When seeds or seedlings were not assigned to the expected mothers, presumably because of secondary dispersal, we did not tentatively reassign them.

### Mating system analyses

For each population, we estimated the outcrossing rate (*t*) in four ways. First, we can rely on *F*_(null)_ seeds (Cameroon and DRC) or *F*_(null)_ seedlings (Gabon) obtained from INEST to estimate the outcrossing rate. The outcrossing rate can be calculated from *F*_(null)_ through the relation *t*=(1−*F*_(null)_)/(1+*F*_(null)_) (Fyfe and Bailey, 1951) that assumes that inbreeding results only from selfing, there is no inbreeding depression and that the inbreeding of adults is at equilibrium. However, as we detected strong inbreeding depression so that adults were non-inbred (see below), we used the relation *t*=1−2.*F*_(null)_ that assumes they are in Hardy–Weinberg equilibrium despite selfing because seeds or seedlings resulting from selfing never reach the adult stage.

Second, outcrossing rate per population was also estimated by leading paternity analyses in CERVUS (Marshall *et al.*, 1998). Assigned mothers (see maternity analysis above) were fixed for each offspring and the paternity analysis was conducted using the self-fertilization option. The following simulation parameters were applied to define the confidence level of paternity analysis assignment: 10 000 simulated mating events; all adults of the given population as candidate father plants; individuals typed at a minimum of five loci; 0.5 as the proportion of candidate fathers sampled; genotyping error rate of 0.1. The outcrossing rate was estimated as the number of observed outcrossing events over the total number of tested offspring.

The per-family self-fertilization rate was estimated for each individual of each family using a Bayesian approach implemented in the MSF software (Chybicki, 2013a, 2013b). The method is based on the mixed mating model, with rates of self-fertilization treated separately for each maternal individual. Markov Chain Monte Carlo analyses were run for 1 million cycles with sampling of the parameters every 1000 steps. The per-family selfing rate was then estimated using a burn-in of 10%. The difference of these selfing rates between populations was tested using unpaired *t*-tests.

Finally, the multilocus outcrossing rate (*t*_m_) and the single-locus outcrossing rate (*t*_s_) were estimated using MLTR v.3.2 (Ritland, 2002) from progeny arrays (*N*=58 families for Cameroon, 32 for DRC and 23 for Gabon). Mating among relatives (biparental inbreeding) was estimated by the difference (*t*_m_−*t*_s_). In case of biparental inbreeding, it is expected that (*t*_s_)<(*t*_m_), and the difference is a minimum estimate of the apparent selfing because of biparental inbreeding (Shaw *et al.*, 1981). Standard deviation of these estimators was evaluated through a bootstrap procedure (1000 repetitions).

### Spatial structure of pollen pools

Relying on mapped mother–offspring genotypic data, contemporary pollen dispersal was inferred using the so-called KINDIST and TWOGENER methods as implemented in the software POLDISP (Robledo-Arnuncio *et al.*, 2007). We followed recommendations from POLDISP's user manual in the preparation of the data sets per population: no mismatch between mother and offspring (loci presenting mismatches were transformed as missing data in offspring), no missing data for the mothers, no seeds resulting from selfing (we used the results from the CERVUS analysis to removed selfed seeds) and a minimum of two offspring per mother. We first used KINDIST to estimate the correlation of paternity of outcrossed progenies within families and between maternal families (separately for each pair of families) from the mapped genotypes of mother–offspring data. The mean number of effective pollen donors (*N*_ep_) that participate to cross-pollination was estimated from the within-sibship correlated paternity (*r*_p_) as *N*_ep_=1/*r*_p_. For comparison (*r*_p_) was also calculated with MLTR (Ritland, 2002) using as input files all correctly-assigned-to-mother offspring (including those resulting from selfing).

To test whether the among-sibship correlated paternity was inversely correlated with the distance between mother trees, we used a Mantel test procedure as implemented in the zt software (Van de Peer, 2002). The slope was negative and significant in each population. We then tested the fit of our data with the different pollen dispersal distribution models available in POLDISP (normal, exponential, exponential power). We used 1000 m (Cameroon and DRC) and 2000 m (Gabon) as reference threshold distances to define unrelated pollen pools, because there was no decrease of the among-sibship correlated paternity beyond these threshold distances. For each population, the best dispersal distribution was chosen by comparing the least-square residuals obtained for each of these distributions. Finally, we used TWOGENER to estimate the effective male population density (*D*_Ep_) having as input the pollen dispersal distribution parameters estimated with KINDIST. The ratio (*D*_Ep_/*D*) provides an indication of the proportion of adult trees that have contributed to reproduction as pollen donors within the population for one given year.

## Results

### Genetic diversity and consanguinity

None of the genetic diversity parameters calculated for the adult cohort differed significantly among populations (data not shown). Only the population in Gabon presented differences in genetic diversity among cohorts (for *NA*_E_, *A*_R_ and *H*_E_; see Table 1), but this can be attributed to a sampling effect (low representation of the seed cohort with 23 seeds coming from only 4 mother trees). The (uncorrected) inbreeding coefficient (*F*) was significantly higher than zero in all populations and cohorts. Estimates correcting for the presence of null alleles, *F*_(null)_, showed a decreasing trend from seeds to adults: *F* was slightly lower in seedlings than in seeds and strongly decreased from seedlings to adult stage. In adults, *F*_(null)_ never departed significantly from zero, indicating that they are not inbred. The unpaired *t*-test on observed heterozygosity per individual was significant (*P*<0.05) only between adults and seeds in Cameroon and DRC, and between adults and seedlings in Cameroon. The nonsignificant result in Gabon between adults and seedlings may be due to the low sample size of adult individuals in this population because mean heterozygosity values showed the same trends as in the other populations.

### Fine-scale SGS and inference of gene dispersal distances

**Figure 2:**
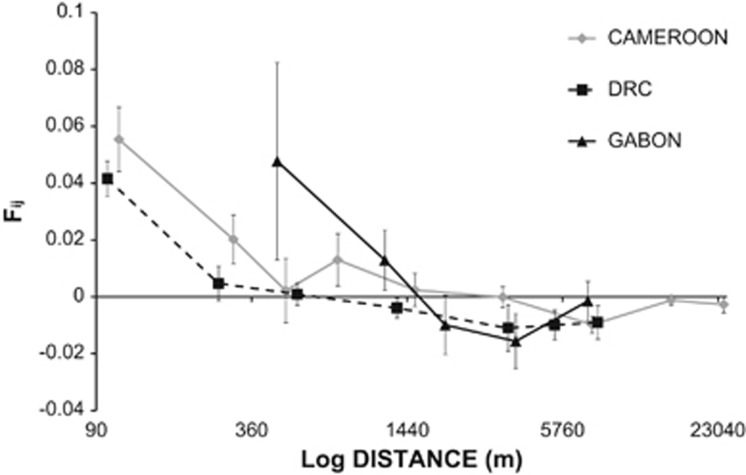
Average kinship coefficients *F*_*ij*_ between pairs of individuals at different geographical distance intervals (log scale) in each population. Vertical bars are s.e.m. The continuous grey line, the continuous black line and the dashed grey line correspond respectively to the Cameroonian, the Congolese and the Gabonese populations.

In all three populations a signal of isolation by distance was observed with pairwise kinship coefficients decreasing significantly with distance (Figure 2 and Table 2). In all populations, kinship for the first distance class (ca. 100 m for Cameroon and DRC, ca. 500 m for Gabon) ranged from 0.04 to 0.06, and quickly dropped with distance, indicating that spatially close individuals are more related. The kinship-distance curve for the low-density Gabonese population was similar to the ones of the medium- to high-density populations but shifted towards larger distances (Figure 2), whereas the *Sp* statistics, which quantify the strength of SGS, were similar for all three populations, ranging from 0.0053 to 0.0073 (Table 2). The procedure to estimate gene dispersal parameters converged only for the Cameroonian population. For *D*_E_=0.820 ind ha^−1^, σ_g_=379±67 m (mean±s.e.), and neighbourhood size Nb=148±54. For *D*_E_=0.410 ind ha^−1^, σ_g_=483±64 m, and neighbourhood size Nb=120±33. No convergence was reached for *D*_E_=0.205 ind ha^−1^.

### Assignment of offspring to mothers

For the Cameroonian population, 384 out of 499 seeds (∼77%) were assigned to the expected mother. In this population, we did not tentatively assign collected seedlings to mothers. For the Congolese and Gabonese populations, respectively 200 out of 254 seeds and seedlings (∼79%), and 179 out of 199 seeds and seedlings (90%) were assigned to the expected mother.

### Mating system analyses

All three populations of *E. suaveolens* presented a mixed mating system (mixtures of selfed and outcrossed pollination). We found a linear trend between selfing rate and population density with one of the methods used (MSF software, Table 2), which was contrary to our expectations as selfing increases with increasing population density. However, the unpaired *t*-test between populations (based on the per-family self-fertilization rates) demonstrates that estimates of outcrossing rates are only significantly different between the Gabonese (*t*_m_=0.850) and Cameroonian (*t*_m_=0.782) populations. This is potentially only because of a methodological bias as the selfing rate could have been underestimated in the population from Gabon where seedlings had to be used instead of seeds. The Gabonese population also presented a higher biparental inbreeding signal as measured by (*t*_m_−*t*_s_) than the two other populations.

### Spatial structure of pollen pools

In all three populations, the among-sibship correlated paternity was negatively and significantly correlated with distance between mother trees (Supplementary Material 4 online). MLTR and POLDISP provided similar estimates of correlated paternity within maternal sibships (*r*_p_) for each population (Table 3). The differences between populations were not significant (*P*>0.05 for all comparisons, unpaired *t*-test). Despite a more than twofold higher population density (*D*) in Cameroon than in DRC, this latter population presented only a slightly lower proportion of individuals participating in pollination (*D*/*D*_Ep_) (Table 3). However, the number of effective pollen donors (*N*_EP_) was higher in the Congolese population than in the Cameroonian one. The Gabonese population presented the lowest *D*_E_ and *N*_EP_. In the absence of confidence intervals around these measures, we were not able to test whether differences were statistically significant. The best fit of the dispersal distribution was obtained with the exponential dispersal kernel in all three populations. The average pollen dispersal distance estimates decreased from 1001 m in the low-density population to 195 m in the high-density population (Table 3).

## Discussion

We analysed patterns of genetic diversity and structure, mating system and gene flow in three populations of the African timber tree species *E. suaveolens*. The three populations present different population densities (for trees with DBH >30 cm). The study of the relationship between contrasting population densities and reproductive biology or genetic diversity parameters can be used to predict one aspect of the potential impact of logging. The Cameroonian population presented the highest density followed by the population from DRC (ca. 40% of the Cameroonian one; Table 3) and finally the population from East Gabon with the lowest density (ca. 6% of the Cameroonian one). Importantly, these results concern only *E. suaveolens* and not *E. ivorense*, both commonly named as tali, but that actually correspond to two clearly differentiated parapatric species (Duminil *et al.*, 2010).

### Lower population density is not associated with lower genetic diversity

No relationship between genetic diversity and population density is supported by our results. Levels of genetic diversity are equivalent across the three populations and across cohorts within each population (Table 1). A previous study indicated that the Cameroonian and the Gabonese populations are located in two different gene pools with equivalent levels of genetic diversity (Duminil *et al.*, 2013). Our data, acquired at a fine-geographical scale, support this result, highlighting that historical factors did not lead to different levels of genetic diversity. Estimates of genetic diversity of the seed cohort also depend on pollen dispersal distance (pollen diversity). The larger the pollen distance, the greater the degree seeds will ‘capture' the pollen diversity of surrounding individuals. We have demonstrated that pollen dispersal distance is inversely related to population density. This indicates that the absence of relationships between population density and genetic diversity in the case of *E. suaveolens* actually corresponds to a drift/migration balance: local genetic drift would be expected to be higher in the low-density population (the strength of drift is inversely proportional to population size), but it is prevented by long-distance pollen dispersal (see below), maintaining genetic diversity.

Nevertheless, we need to be careful about extrapolation of such a conclusion. Despite the absence of a relationship between genetic diversity and population density in this study and others (Cloutier *et al.*, 2007; Fageria and Rajora, 2013), it is not certain that genetic diversity is secure. Indeed, long-term impacts of selective logging across multiple cutting cycles investigated through modelling have led to detrimental effects (Degen *et al.*, 2006; Sebbenn *et al.*, 2008; Wernsdörfer *et al.*, 2011; but see Vinson *et al.*, 2014). One of these models particularly emphasized the impact of juvenile mortality on genetic diversity (Wernsdörfer *et al.*, 2011). We do not have specific data on *E. suaveolens* juvenile mortality, but the population structure is clearly truncated at small-diameter classes in the two nonlogged populations (Cameroon and DRC; Supplementary Material 1 online), indicating the poor regeneration capacity of the species under closed canopy as expected for light-demanding species. This suggests that the future replacement of seed trees after logging is not ensured at the same level, unless intensive enrichment planting is carried out as done by some FSC-certified logging companies of Central Africa. This effect has to be carefully modelled before drawing any clear conclusion on the long-term impact of logging on *E. suaveolens* genetic diversity. Further information on the impact of cutting on species' regeneration is needed to propose recommendations. *E. suaveolens* is a light-demanding species in its early stages and opening the canopy by logging might have a positive influence on its regeneration (Kouadio, 2009) if the openings are large enough. Moreover, important parameters such as cutting diameter, growth rates, natural mortality rate and time between two cutting cycles have to be integrated for accurate interpretation of results.

### Lower population density is not associated with stronger spatial genetic structure

The strength of SGS depends on a migration–drift equilibrium. Overall, the strength of *E. suaveolens* SGS as measured by the *Sp* statistic is typical of tree species (Table 2; Vekemans and Hardy, 2004) and suggests that seed-mediated dispersal is relatively efficient in this species (Dick *et al.*, 2007). It is expected that lower-density populations present stronger SGS as local drift would be more important (Vekemans and Hardy, 2004). Here, we can only test this relationship between the population from Cameroon and the population from DRC as the SGS estimation for the population from Gabon lacks precision because of a small sample size. The population from DRC does not seem to present a stronger SGS than the population from Cameroon, suggesting that the strength of SGS is not related to population density (large overlap of s.e.m. values; Table 2). This suggests that the potentially larger drift effect expected at lower densities is compensated by increased dispersal distance (see below). Such a pattern has already been observed in another African tropical tree species, *Aucoumea klaineana* (Born *et al.*, 2008).

### Lower population density is not associated with higher selfing rate and inbreeding

There is no clear relationship between population density and mating system at least for the range of densities observed in the current study (Table 2). The only significant difference, between the lowest and the highest population density (between Gabon and Cameroon) is potentially only due to a methodological bias. Altogether, all three populations seem to present similar levels of selfing, regardless of population density (between 13 and 19% based on MLTR analyses; Table 3). The same result has already been obtained for a set of timber species, where the selfing rate has been demonstrated to remain unchanged after selective logging (Cloutier *et al.*, 2007; Lourmas *et al.*, 2007; Ojeda-Camacho *et al.*, 2013). However, in these studies, the outcrossing rate was close to one that tends to support the existence of a strong auto-incompatibility system. Here, we also observed similar levels of selfing despite different population densities. The stability of this mixed mating system is puzzling. It has been proposed that the evolutionary stability of mixed mating systems is determined by the timing and relative amount of self- and outcross-pollination (Holsinger, 1991). In other words, if outcross pollen supply is limited, the selfing rate will be higher. We can expect higher pollen limitation in low-density populations, but this is probably not the case for *E. suaveolens*, as pollen distance increases with decreasing density (see below). The compensation of population density reduction by larger pollen flow would explain that selfing rate is relatively stable in populations that present different levels of density. Alternatively, the relative stability of the mixed mating system could be explained by post-pollination mechanisms, such as partial self-incompatibility systems (Goodwillie *et al.*, 2005).

Like most tree species, *E. suaveolens* is subject to inbreeding depression. Indeed, we observed a trend of decreasing inbreeding from seed to adult stage (Table 1), suggesting that individuals resulting from selfing die before reaching the adult stage (Barrett and Charlesworth, 1991). This trend was significant only for two out of the three populations (Cameroon and DRC). Selfing is generally detrimental in tree species because of their sensitivity to inbreeding depression, a likely consequence of their long generation time (Charlesworth, 2003; Duminil *et al.*, 2009). This expression of inbreeding depression has important consequences in terms of species population dynamics in relation to selective logging. Adult individuals that become genetically isolated after logging will have a very low probability of producing viable descendants. Though we have demonstrated that selfing rate is somewhat independent from population density, studying the relationships between degree of genetic isolation associated with greater distances than observed in the current study and mating system/seed set of trees in low-density populations of *E. suaveolens* will be necessary to further test the existence of this detrimental effect.

Biparental inbreeding seems to be more pronounced in the low-density population than in the high-density one (Table 2). The main cause of biparental inbreeding might be the low efficiency of seed-mediated gene flow as we demonstrated that pollen-mediated gene flow decreases with population density. If distance of seed dispersal is similar (and low) in all three populations, it is expected that the lowest density population is also the one most prone to biparental inbreeding. Here we did not find any clear relationship between population density and level of SGS, which tends to contradict this hypothesis. However, the absence of an accurate estimation of SGS for the low-density population due to small sample size limits our capacity for interpretation. Alternatively, the presence of a biparental inbreeding signal could suggest preferential mating between related individuals as would be the case if phenological asynchrony has a genetic origin. In the absence of phenological data, we cannot further interpret our results.

### Lower population density is not associated with fewer pollen donors contributing to the pollination of each mother tree

Levels of correlated paternity within maternal sibships are similar in all three populations (Table 3). This result is well in line with an increase in the average distance of pollen dispersal with decreasing density and further provides information on the quality of mating events (similar number of pollen donors in different population densities). One can expect that this qualitative estimation can also indicate the quantity of pollen received, but this is actually not necessarily the case. The estimations that have been obtained in this study provide a picture of mating events based only on collected seeds or seedlings. These observations are independent from the actual level of seed production. We can obtain similar patterns of correlated paternity in the presence or in the absence of pollen limitation (with respectively low and high seed production). Measures of seed production through direct observations at different classes of physical isolation to the nearest neighbour need to be obtained to get a clearer view of the quantitative aspects of mating events.

Our results suggest that the effective density of pollen donors might be more reduced in low-density populations than in the high-density ones. This result needs to be interpreted with caution as we were not able to test the statistical significance of this trend. This result is, however, in line with our expectation and with other studies (Mimura and Aitken, 2007; Tani *et al.*, 2012; Masuda *et al.*, 2013).

### Lower population density is associated with a change of pollen dispersal distances

The extent of gene flow is correlated with population densities as pollen dispersal distances have been shown to decrease with population density. The average distance of pollen dispersal varies from 200 m (Cameroon) to 1000 m (Gabon; Table 3). In other words, these results suggest that at lower population densities, trees are still connected as a result of larger pollen dispersal distances. If pollinating species are identical in all three populations, this can outline their capacity to travel longer distances when flowering trees are more distant. Increased pollen dispersal distances in the case of lower tree densities have already been reported for other tropical species (White *et al.*, 2002; Dick *et al.*, 2003; Born *et al.*, 2008). This pattern probably explains the absence of relationships between population density and previously discussed factors (genetic diversity, SGS, mating system, inbreeding depression) within *E. suaveolens*.

### Logging implications

Norms differ among the three studied countries on the minimum cutting diameters. Logging intensity of *E. suaveolens* is theoretically lower in Gabon (minimum cutting diameter of 70 cm) than in Cameroon and DRC (minimum cutting diameter of 50 cm). Our results suggest that such logging intensities in populations with the same characteristics as those studied in Cameroon and DRC would not affect a population's viability in a single cutting cycle. Given logging norms in these countries, we can estimate that population density of adult individuals would be reduced to 0.47 and 0.30/ha respectively in Cameroon and DRC. Such population densities are still higher than the population density in Gabon. However, models need to be developed to investigate the impact of multiple cutting cycles. It is difficult to predict the impact of logging on the population from Gabon as we have no indication of pollen dispersal distances at lower densities. This study needs to be completed by additional data on pollen limitation, notably through the investigation of the relationships of seed set, fruit phenology, seed–tree distance to the nearest neighbour and selfing rate. We recommend conducting such a study in populations that present densities equivalent to or lower than the population from Gabon. Such studies require exhaustive sampling, detailed cartography of the population structure and long-term monitoring of the flowering and fruiting phenology, which are challenging to organize in the field.

## Conclusion

The comparison of three different populations with contrasting densities of the timber species *E. suaveolens* provides indications of the relationships between population density, mating pattern and genetic diversity. Equivalent levels of genetic diversity, selfing rates and correlated paternity suggest that these population parameters are resilient to a decrease of population density, one of the key consequences of selective logging. In particular, although the species exhibits inbreeding depression, there is no evidence of a loss of quality of the seed produced at low densities. This stability can be explained by the negative relationship observed between pollen dispersal distances and population density, a phenomenon that may be prevalent in plant populations. Although these results are reassuring regarding the risk that selective logging may impede the regeneration of timber tree populations, our study did not allow us to test whether seed production was affected by a decrease in density, nor to address all the other consequences of logging, notably those related to habitat perturbations, which were not the focus of the study. Additional work is thus necessary to provide logging recommendations, such as the definition of a maximum distance to be kept between seed trees to allow the qualitative and quantitative reproduction of the species. We suggest that investigations need to be conducted in low-density populations, which are more threatened by population reduction.

## Data Archiving

Genotype data available from the Dryad Digital Repository: http://dx.doi.org/10.5061/dryad.b78fb.

## Figures and Tables

**Table 1:** Parameters of genetic diversity of the different cohorts of the *Erythrophleum suaveolens* populations

*Population*	*Cohort*	N	NA_*E*_	A_*R*_	H_*E*_	H_*O*_	F	F_*(null)*_
Cameroon	Adults	177	3.63	5.03	0.628	0.531	0.155	0.003
	Seedlings	162	3.53	4.94	0.606	0.488	0.195	0.122
	Seeds	499	3.51	5.02	0.624	0.498	0.202	0.157
Difference among cohorts^a^			NS	NS	NS	NS*	NS	—
DRC	Adults	88	3.39	5.07	0.581	0.512	0.119	0
	Seedlings	13	3.07	4.38	0.535	0.465	0.130	NA
	Seeds	238	3.30	4.95	0.590	0.470	0.203	0.110
Difference among cohorts^a^			NS	NS	NS	NS*	NS	—
Gabon	Adults	31	4.11	6.08	0.658	0.515	0.217	0
	Seedlings	175	4.51	5.98	0.648	0.489	0.246	0.116
	Seeds	23	3.19	4.68	0.600	0.461	0.232	0.132
Difference among cohorts^a^			*	***	*	NS	NS	—

Abbreviations: *A*_R_, Allelic richness (*k*=24); DRC, Democratic Republic of Congo; *F*, Inbreeding coefficient (potentially biased by null alleles); *F*_(null)_, Corrected inbreeding coefficient under a population inbreeding model (unbiased by null alleles); *H*_E_, Gene diversity corrected for sample size; *H*_O_, Observed heterozygosity; *N*, Sample size; NA, not available; *NA*_E_, Effective number of alleles; NS, not significant.

a Differences of genetic diversity among cohorts as tested through a two-way analysis of variance (ANOVA) procedure (*P<0.05; ***P<0.001); NS* indicates test that were not significant with the two-way ANOVA but that were significant using the paired *t*-test (see text for details); ‘—' indicates that no test was done for the corresponding parameter as estimates of *F*_(null)_ per loci cannot be estimated.

**Table 2:** Mating system and fine-scale spatial genetic structure parameters of the *Erythrophleum suaveolens* populations

	*Cameroon*	*DRC*	*Gabon*
*F*_1_ (mean±s.e.)^a^	0.0554* (0.0113)	0.0415* (0.0062)	0.0477* (0.0347)
*Sp* statistic (mean±s.e.)	0.0061 (0.0016)	0.0053 (0.0012)	0.0070 (0.0081)
*t* (*F*_(null)_)^b^	0.690	0.780	0.740
*t* (CERVUS)^c^	0.800 (0.166–1.00)	0.830 (0.571–1.00)	0.780 (0.428–1.00)
*t*_m_^d^ (s.d.)	0.782 (0.103)	0.818 (0.145)	0.850 (0.177)
*t*_m_^e^ (s.d.)	0.816 (0.030)	0.885 (0.029)	0.874 (0.046)
*t*_m_–*t*_s_^f^ (s.d.)	0.098 (0.020)	0.109 (0.023)	0.161 (0.033)

Abbreviation: DRC, Democratic Republic of Congo.

Mating system parameters are based on the genotypes of seeds (Cameroon and DRC) or seedlings (Gabon), whereas fine-scale spatial genetic structure estimates are based on the genotypes of adults.

a *F*_1_: mean kinship coefficient between individuals at the first distance class.

a *Significant *F*_1_ values (*P*<0.001).

b Outcrossing rate *t* as calculated through the relation *t*=1−2*F*_(null)_.

c *t* as estimated through a paternal analysis in CERVUS, numbers in brackets refer to the range of *t* observed in the population using progeny array with a minimum size of six offspring.

d *t*_m_: multilocus population outcrossing rate (MSF).

e *t*_m_: multilocus population outcrossing rate (MLTR).

f *t*_m_−*t*_s_: indirect estimation of the presence of biparental inbreeding (MLTR).

**Table 3:** Parameters of pollen dispersal in each population

	*Cameroon*	*DRC*	*Gabon*
*D*: density of adults per ha (diameter >30 cm)^a^	1.72	0.68	0.11
*D*_Ep_: estimated effective density of pollen donors per ha^b^^,^^c^	0.40	0.13	0.01
*D*_Ep_/*D*: proportion of individuals that participate to the pollination (%)	23.3	19.1	9.0
Global φ_ft_^b^^,^^c^	0.061	0.059	0.094
*r*_p_: average within-sibship correlated paternity^d^ (*N*: sample size/s.e.)	0.112 (58/0.045)	0.087 (32/0.051)	0.164 (23/0.057)
*r*_p_: average within-sibship correlated paternity^e^ (*N*: sample size/s.e.)	0.110 (57/0.034)	0.084 (28/0.039)	0.153 (22/0.044)
*N*_EP_: number of effective pollen donors^c^	9	12	7
Average pollen dispersal distance (in m)^e^	195	346	1001

Abbreviation: DRC, Democratic Republic of Congo.

a As measured in the field.

b Obtained by TWOGENER.

c Confidence intervals cannot be derived for these estimators.

d Obtained by MLTR.

e Obtained by KINDIST using an exponential dispersal distribution model.
