# Supplementary material for: Relationships between population density, fine-scale genetic structure, mating system and pollen dispersal in a timber tree from African rainforests
Source: Heredity (Edinb). 2015 Dec 23;116(3):295–303. doi: 10.1038/hdy.2015.101 (PMC4806568; doi:10.1038/hdy.2015.101)
Supplement: Supplementary Information [file hdy2015101x1.docx]

***Supplemental online material 1:*** Structure of the three populations.


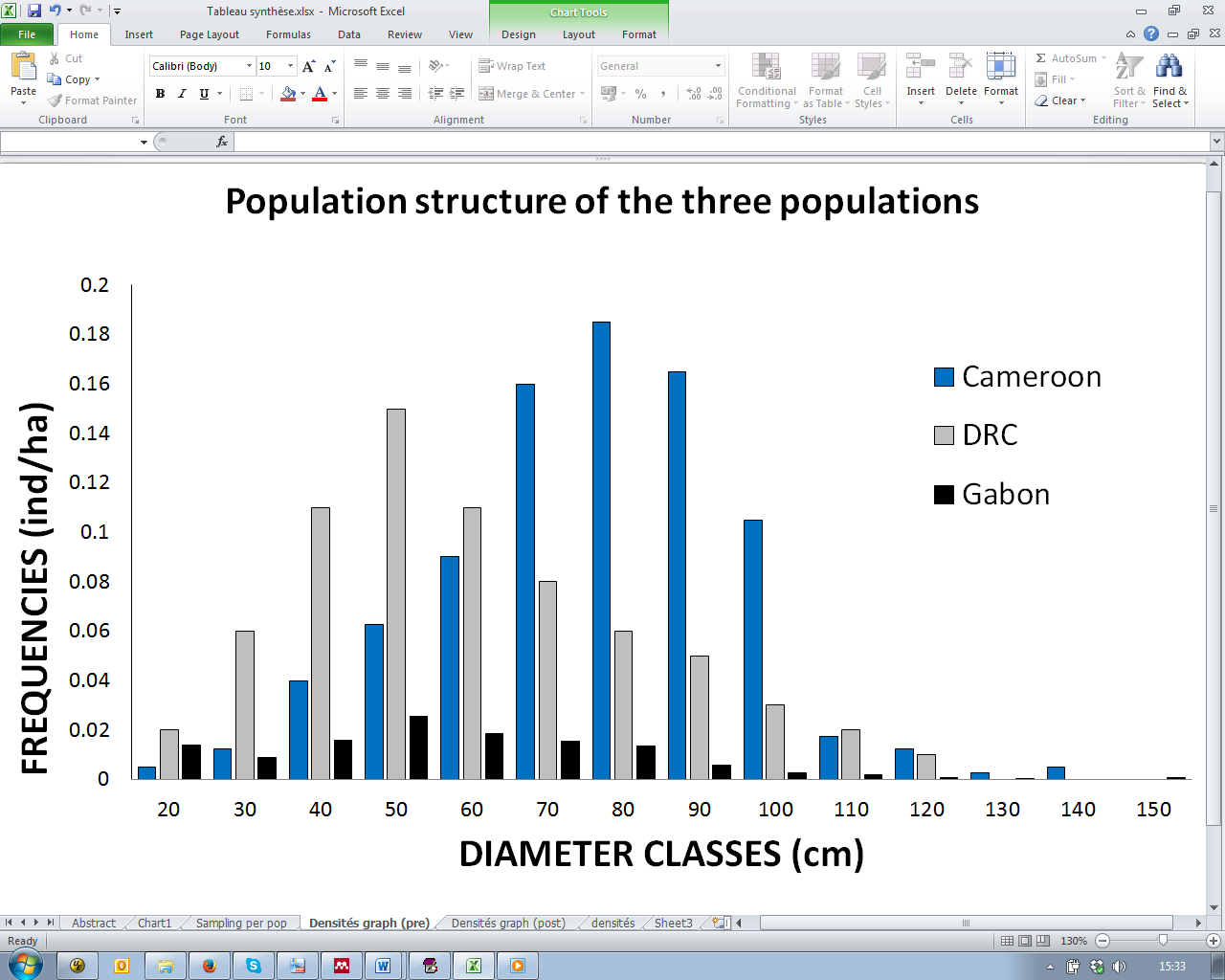


***Supplemental online material 2:*** Sampling characteristics in each population.

|  |  | **Adult trees** | **Adult with offspring** | **Seedlings** | **Seeds** |
| --- | --- | --- | --- | --- | --- |
| **Cameroon** | Central Zone | 47 | 24 | 86 | 164 |
|  | Along transects | 130 | 48 | 76 | 335 |
| **DRC** | Central Zone | 63 | 9 | 13 | 75 |
|  | Along transects | 25 | 24 |  | 163 |
| **Gabon** | Large Zone | 31 | 23 | 175 | 23 |

***Supplemental online material 3:*** Distribution of the number of analysed offspring (seeds and/or seedlings) per family


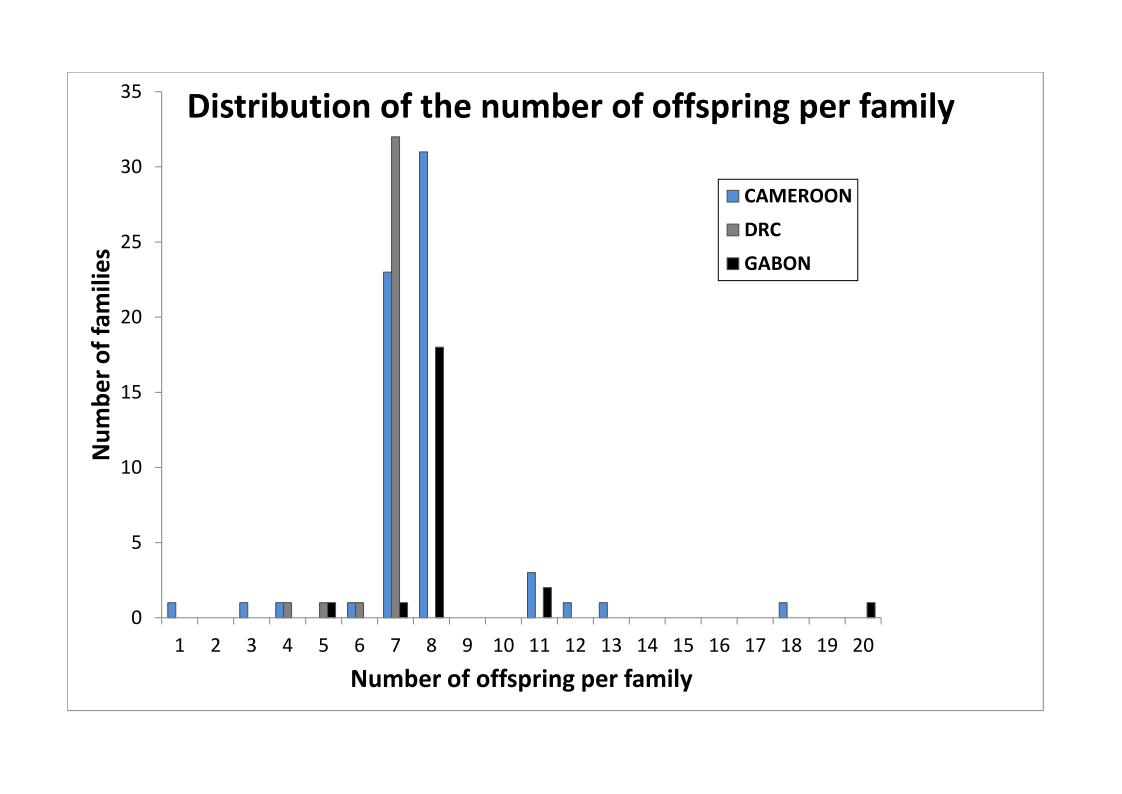


***Supplemental online material 4:*** Relationships between correlation of paternity and distance between mothers in all three populations. Grey dots correspond to among-sibship correlated paternity and black dots correspond to within-sibship correlated paternity. The correlation coefficient *r* as well as the associated *P* values (as obtained through a Mantel test procedure) are indicated in each graph.


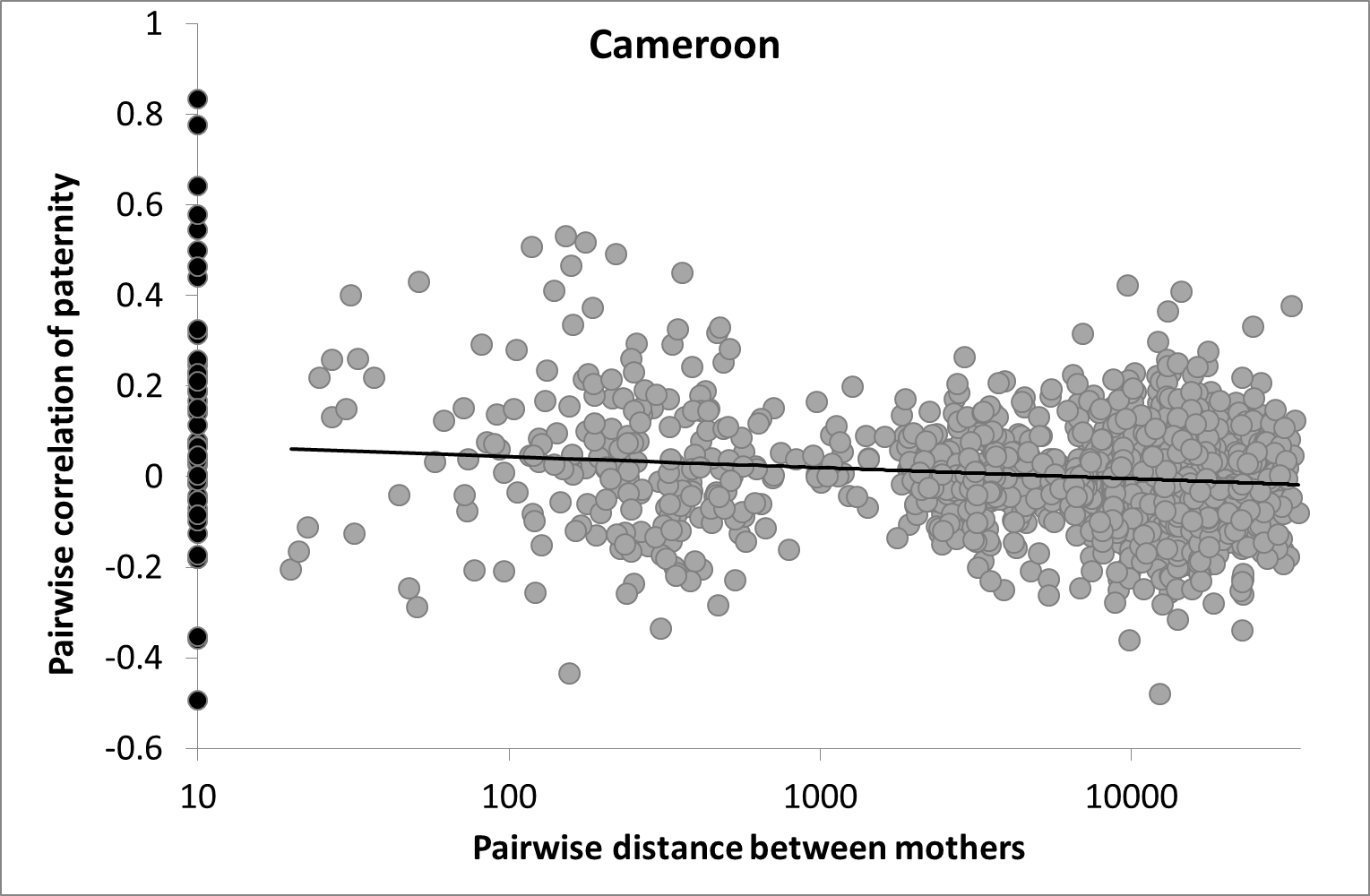


**r = -0.146 ; P < 0.001**


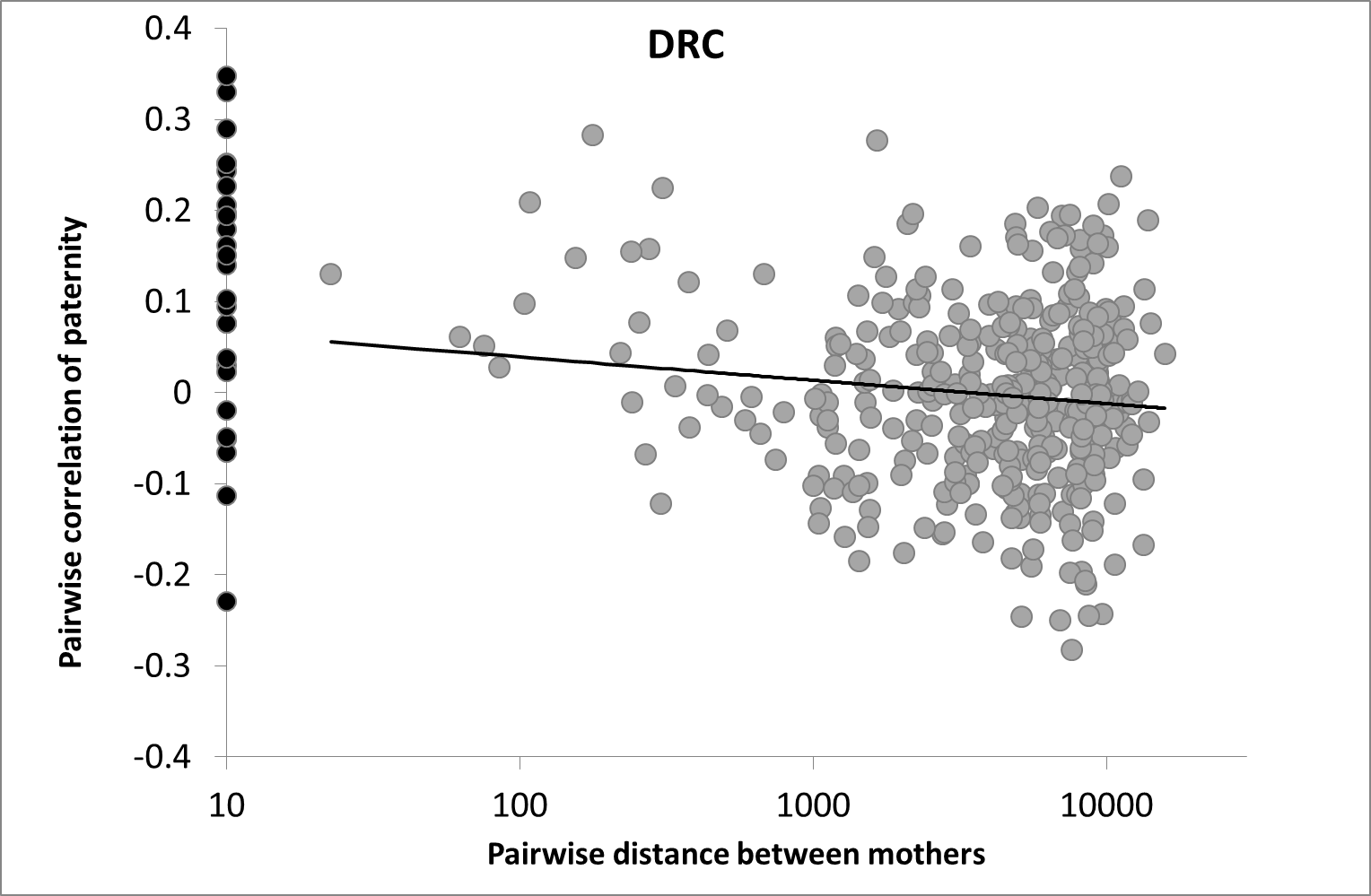


**r = -0.118 ; P < 0.020**


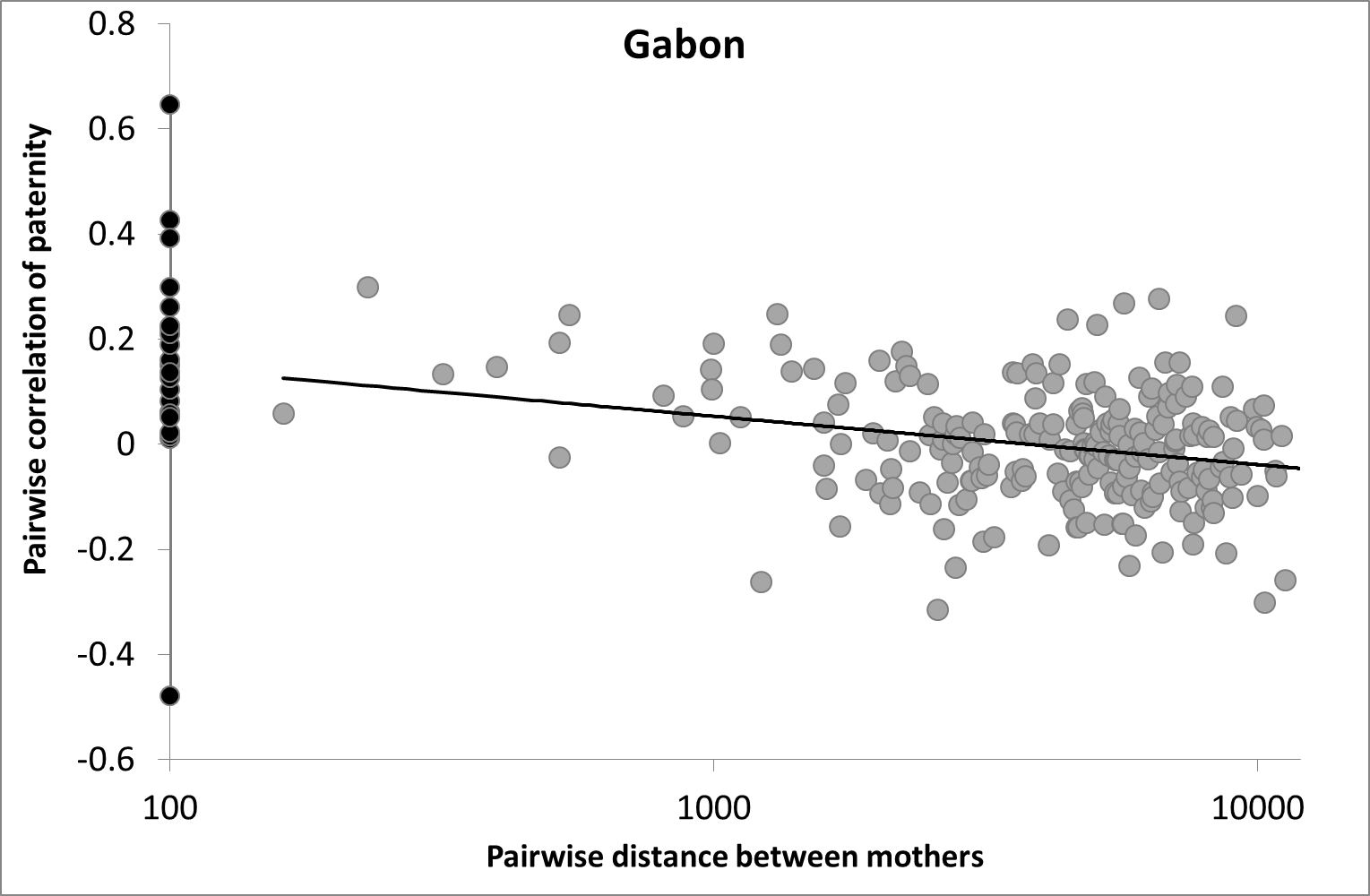


**r = -0.264 ; P < 0.001**
